# Dental Surgical Activity in Hospitals during COVID-19: A Nationwide Observational Cohort Study

**DOI:** 10.1177/23800844231216356

**Published:** 2024-01-03

**Authors:** J. Booth, A.J. Fowler, R. Pearse, P. Dias, Y.I. Wan, R. Witton, T.E.F. Abbott

**Affiliations:** 1 Dental Public Health and Primary Care, Barts & The London School of Medicine and Dentistry, Queen Mary University of London, London, UK; 2 Peninsula Dental School, University of Plymouth, Drake Circus, Plymouth, UK; 3 Critical Care and Perioperative Medicine Research Group, William Harvey Research Institute, Queen Mary University of London, London, UK

**Keywords:** dental public health, oral and maxillofacial surgery, hospital dentistry, health services research, cohort studies, oral surgery

## Abstract

**Introduction:**

The number of surgical extractions performed in hospitals in England remains unclear. This study reports the volume of surgical extractions conducted in hospitals and change in activity during the COVID-19 pandemic.

**Methods:**

We conducted a nationwide observational cohort study using Hospital Episode Statistics (HES) in England for patients undergoing surgical removal of a tooth (defined using OPSC-4 code F09) between April 1, 2015, and December 31, 2020. Procedures were stratified by age, gender, and urgency (elective or nonelective), reported using descriptive statistics, number, and percentage. We conducted post hoc modeling to predict surgical activity to December 2023. In addition, we contrasted this with aggregate national data on simple dental extraction procedures and drainage of dental abscesses in hospital as well as dental activity in general practice.

**Results:**

We identified a total of 569,938 episodes for the surgical removal of a tooth (females 57%). Of these, 493,056/569,938 (87%) were for adults and 76,882/569,938 (13%) children ≤18 years. Surgical extractions were most frequent in adult females. Elective cases accounted for 96% (*n* = 548,805/569,938) of procedures. The median number of procedures carried out per quarter was 27,256, dropping to 12,003 during the COVID-19 pandemic, representing a 56% reduction in activity. This amounted to around 61,058 cancelled procedures. Modeling predicts that this activity has not returned to prepandemic levels.

**Conclusions:**

The number of surgical extractions taking place in hospitals during the pandemic fell by 56%. The true impact of this reduction is unknown, but delayed treatment increases the risk of complications, including life-threatening infections.

**Knowledge Transfer Statement:**

The result of this study provides an evidence-based overview of the trends relating to surgical extractions of teeth in England taking place in hospitals. This information can be used to inform service and workforce planning to meet the needs of patients requiring surgical extractions. The data also provide an insight into the oral health needs of the population in England.

## Introduction

Dental care in England is predominantly delivered in community-based settings by general dental practitioners (GDPs). Patients are referred to hospital when they require care beyond the expected competencies of a GDP. Oral surgery patients are referred for surgically challenging extractions or if the patient is medically compromised (NHS England 2015). Some referrals are due to the requirement of conscious sedation or a general anesthetic to facilitate care (Goldthorpe et al. 2018). Most hospitals can also admit patients who present at emergency departments requiring urgent treatment or inpatient care such as those with severe dental abscesses, but the exact number and change in trends for these admissions remains uncertain.

The COVID-19 pandemic changed the delivery of dentistry globally, causing large-scale disruption to dental services. Dentistry was particularly affected due to the risks of COVID-19 transmission from aerosols produced during dental treatment (Amante et al. 2021), redeployment of dental staff (Sacoor et al. 2020), and the postponement of nonurgent surgery in hospital settings to prioritize the care of those with COVID-19 (Al-Jabir et al. 2020). On March 25, 2020, routine dental care in England was suspended. Urgent dental care (UDC) sites were established to provide face-to-face care where clinically necessary (Appendix 1). It was not until June 8, 2020, that dental practices began to resume the delivery of care when personal protective equipment (PPE), infection control, and patient prioritization guidance were available (Hurley et al. 2020). This hiatus was accompanied by the suspension of routine surgery in hospital settings and a focus on the management of urgent cases (Glasbey et al. 2022; Moore et al. 2022). Dental services remained impacted through subsequent lockdowns, and the longer-term impacts of the pandemic are now being seen with increased National Health Services (NHS) waiting times (Ehsan 2022; Westgarth 2021a). Surgically, in hospitals following the suspension of elective surgeries, there was a phased return with a reduced capacity due to staff sickness, isolation guidance, reduced operating theater capacity, and new infection control protocols, including the introduction of fallow time after aerosol-generating procedures (AGPs) (O’Donnell et al. 2022). While this study focuses on the provision of dental care in England, similar levels of disruption and impacts on the delivery of dental care were seen across the globe.

Over a third of adults in England required dental treatment or advice during the time services were limited (Office for Health Improvement & Disparities 2022) but had difficulty accessing care. It is estimated that 19 million fewer dental treatments were delivered due to restrictions imposed during the pandemic (British Dental Association 2020). However, the exact number of dental procedures that were cancelled because of COVID-19 in hospital settings is unknown. Case studies emerged suggesting that the cessation of routine dentistry had contributed to an increase in the number of patients requiring urgent dental treatment for life-threatening dental conditions, such as Ludwig’s angina (Altıntaş 2022), but there are no objective studies to support this hypothesis.

The primary aim of this study was to report the number of surgical extractions that occur in hospitals and the impact of the pandemic on this procedure. Furthermore, the study aims to outline trends seen in admissions for simple extractions and for the drainage of dental abscesses.

## Methods

### Ethics Approval

This cohort study analysis was approved by the Health Research Authority (20/HRA/3121) and the NHS Digital Independent Group Advising on the Release of Data (DARS-NIC-375669-J7M7F).

### Study Design and Setting

This was a national observational cohort study including all hospital admissions for surgical extractions of teeth in hospital settings in England between April 1, 2015, and December 31, 2020. Data for the COVID-19 pandemic included admissions from January 1, 2020, to December 31, 2020. Data from April 1, 2015, to December 31, 2019, were used as a pre–COVID-19 comparator. Methods were reported in line with the Strengthening the Reporting of Observational Studies in Epidemiology (STROBE) checklist.

National aggregate data describing dental activity in general practice were included to provide a context for the surgical extraction data. These data are presented by financial year and extracted for the years 2015–2016 through 2020–2021. Data extracted were volume and type of courses of treatment completed. Additional data on hospital admissions for the “simple extraction of a tooth” and “drainage of abscess of alveolus of tooth” were included.

### Data Sources

The study used Hospital Episode Statistics for Admitted Patient Care (HES-APC), which outlines every episode of hospital care in the NHS in England but not those seen in a private fee-paying capacity. This data set also included NHS-funded treatments performed in the private sector, during the COVID-19 pandemic. NHS England signed a contract to use private facilities to increase capacity (NHS England 2020). The database includes information relating to patient demographics, age, sex, ethnicity, diagnostic data, and multiple deprivation index (IMD) alongside procedural information such as admission category and the length of stay. The database also includes procedural information as recorded using Office of Population Censuses and Surveys classification of interventions and procedures version 4.7 (OPCS-4.7) codes as described previously (NHS England 2020; Abbott et al. 2017; Fowler et al. 2019; Abbott et al. 2021; Fowler, Dobbs, et al. 2021; Fowler et al. 2022). These codes usually define procedures that are performed in an operating theater and/or under general anesthesia.

Data relating to hospital admissions for the “simple extraction of a tooth” or “drainage of abscess of alveolus of tooth” were extracted from public aggregate data available from NHS Digital. Data relating to dental activity in primary care were taken from NHS Dental Statistics (NHS Digital 2022). The dental activity in primary care was stratified by treatment band. Treatment bands are used by the NHS dental system, and each band corresponds to a specific patient charge and remuneration for the provider. As an example, band 1 treatments include clinical examinations; band 2, procedures such as fillings and dental extractions; and band 3, treatments such as bridges, crowns, and dentures. The urgent band includes an exam and any treatment needed to resolve a patient’s dental pain or stabilize disease (NHS England 2021). Data relating to the dental workforce were extracted from NHS Digital for the number of dentists with NHS activity practicing during each financial year.

### Study Population

All patients in England undergoing surgical procedures for “surgical removal of a tooth” as determined by OPCS-4.7 code F09 during the duration of the study were included. No exclusion criteria were applied to age, sex, or ethnicity. Prescence of F09 in any OPCS code field in HES-APC was sufficient for inclusion in the study. For the supplemental data, all patients undergoing a surgical procedure for “simple extraction of a tooth” as determined by OPCS-4.7 code F10 and “drainage of abscess of alveolus of tooth” code F16.1 were included.

### Outcomes

The primary outcome measure was the number of hospital admissions for a surgical removal of a tooth. The secondary outcome measure was the number of hospital admissions associated with simple removal of a tooth or drainage of abscess of the mouth.

### Data Processing

All hospital episodes associated with OPCS-4.7 code F09 were selected from finished consultant episodes (FCEs), captured using a combination of the FCE and episode status (EPISTAT) fields. We excluded records with missing admission method, patient sex, maternity admissions, and admissions with implausible procedure dates. For each hospital episode, the procedure was categorized as either elective or nonelective using admission methods. IMD was mapped using patient lower super output area to the relevant decile of deprivation from 2019. The data were aggregated by sex, age (18 and older, or under 18), category of procedure (elective or nonelective, as described in Appendix 2), ethnicity, IMD, month, and year of the procedure. Secondary outcomes included the length of stay. We analyzed at the level of the hospital admission and selected the first episode per admission per patient. This means that patients could be included multiple times if they had multiple admissions.

### Statistical Analysis

Descriptive statistics were used to describe the volume of surgical extractions performed during the historical comparative period and during the COVID-19 pandemic with confidence intervals calculated on Stata version 17 (StataCorp). The median quarterly frequency of surgical extractions from quarter 1 (2015–2016) to quarter 3 (2019–2020) was calculated as the surgical activity during the historic comparative period. The same approach was then used to determine the median for surgical extractions per quarter during the COVID-19 pandemic, defined as quarter 4 (2019–2020) to quarter 3 (2020–2021). These were then compared to provide an estimated reduction in activity as a percentage of prepandemic levels and to quantify the deficit in surgical extractions during the pandemic.

### Estimation of Volume of Surgical Extractions

Our analysis reports observations from analysis of record-level data until December 31, 2020, which was supplemented by publicly available procedure-level data between January 1, 2021, and March 31, 2022. Post hoc modeling was used to estimate the frequency of surgical extractions until December 2023, using the approach of Dobbs et al. 2021. Historic trends were then used to predict surgical extraction activity between 2022 and December 2023. From 2017 to 2019, there was a steady decline in activity prior to COVID-19. An optimistic view was presented that assumed that pre–COVID-19 activity levels were reached by April 2022, with this increasing up to activity levels seen in 2017 by the end of the modeling period. A pessimistic outlook was presented that modeled activity levels based on activity only reaching 75% of prepandemic levels by December 2023. A realist middle ground is presented plotted between these 2 extreme scenarios.

## Results

### Demographics

**Figure 1.**
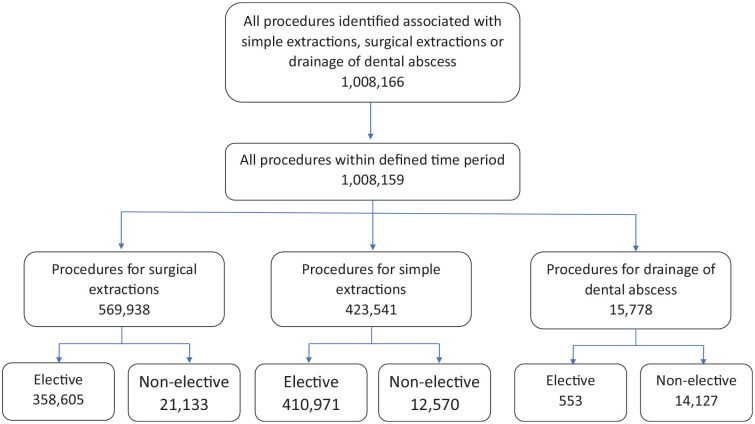
Flow diagram summarizing patient inclusion. Seven cases identified for surgical extractions were excluded due to having only a single data point.

Between 2015 and 2020, 569,938 hospital patient episodes for surgical removal of tooth (confidence interval [CI], 499,553–640,323) were identified (Fig. 1). In total, 548,766 cases (96%) were elective and 21,133 were nonelective cases (4%); full demographics for admissions are outlined in Table 1. In 4,346 (0.7%) of cases, the patient also had a diagnosis of “cellulitis or abscess of mouth” (Table 2). We identified 15,778 admissions for “drainage of abscess of alveolus of tooth” (F16.1) and 428,013 for “simple extraction of tooth” (F10). For the admissions for drainages of dental abscesses, 53.0% were for men and 99.4% were nonelective admissions. The mean age of patients admitted was 36 y, and mean duration of hospital stay was 1.90 d. Admissions for simple extractions of a tooth were predominantly for women (51.1%) and elective admissions (96.0%). The mean age of patients admitted was 26 y, with a median duration of hospital stay of 2.26 d.

**Table 1.** Characteristics of Patients Stratified by Surgical Procedure.

Characteristic	Surgical Extractions (F09)	Simple Extractions (F10)	Drainage of Dental Abscess (F16.1)
*N*	569,938 (CI, 499,553–640,323)	423,541 (CI, 302,338–544,744)	15,778 (CI, 13,346–18,120)
Age			
Mean age	31 (IQR, 24–47)	25.77 (CI, 24.89–26.65)	36.02 (CI, 34.99–37.05)
>18	76,882 (13.49)	240,094 (56.69)	2,249 (15.32)
18+	493,056 (86.51)	185,646 (43.83)	13,481 (91.83)
Sex			
Male	245,882 (43.14)	209,311 (49.42)	8,354 (56.91)
Female	324056 (56.86)	218,585 (51.61)	7,419 (50.54)
Admission category			
Elective	358,605 (62.92)	410,971 (97.03)	553 (3.77)
Nonelective	21,133 (3.71)	12,570 (2.97)	14,127 (96.23)
Length of stay			
Median number of days (range)	2.77 (2.59–3.29)	2.26 (2.03–2.61)	1.90 (1.80–2.06)
Ethnicity			
Black	26,886 (4.7)	—	—
White	376,314 (66)	—	—
Asian	28,340 (5)	—	—
Other	16,376 (2.9)	—	—
Missing	121,939 (21.4)	—	—
Index of multiple deprivation (IMD)			
Unknown	4,116 (0.72)	—	—
1 (most deprived)	61,243 (10.75)	—	—
2	68,840 (12.08)	—	—
3	68,598 (12.04)	—	—
4	62,802 (11.02)	—	—
5	57,996 (10.18)	—	—
6	55,559 (9.75)	—	—
7	51,373 (9.02)	—	—
8	50,300 (8.83)	—	—
9	46,923 (8.23)	—	—
10 (least deprived)	42,105 (7.39)	—	—

Data are presented as *n* (%) unless otherwise stated. Demographic data are presented according to procedure requiring admission. Patients were excluded the analysis for surgical extractions (F09) for age, ethnicity, and IMD if they did not have data related to length of stay. Information relating to the ethnicity and IMD is not stratified by procedure type in national aggregate data from NHS Digital and therefore is not presented for codes F10 and F16.1.

CI, confidence interval; IQR, interquartile range.

**Table 2.** Demographic Comparison of Surgical Extractions Pre–COVID-19 and during the COVID-19 Pandemic.

Characteristic	Pre–COVID-19	COVID
Age	31 (IQR, 24–47)	31 (IQR, 23–47)
Sex		
Male	239,713 (43.2)	6,136 (42.3)
Female	315,620 (56.8)	8,386 (57.7)
Diagnosis of cellulitis of mouth (K12.2)	4,135 (0.7)	211 (1.5)
Admission category		
Elective	534,982 (96.3)	13,784 (94.9)
Nonelective	20,351 (3.7)	738 (5.1)
Ethnicity		
Black	26,121 (4.7)	765 (5.3)
White	367,210 (66.1)	9,104 (62.7)
Asian	27,500 (5)	840 (5.8)
Other	15,833 (2.9)	543 (3.7)
Missing	118,669 (21.4)	3,270 (22.5)
Length of stay (LOS)		
Number of admissions with at least 1-d LOS	12,189 (2.2)	510 (3.5)
Median LOS	0 (IQR, 0–0)	0 (IQR, 0–0)
Total bed days	86,043	3,569

Data are presented as *n* (%) unless otherwise stated. Demographic data are presented for surgical extractions as defined by code F09 and stratified by whether they were undertaken in the pre–COVID-19 comparison period or during the COVID-19 pandemic. Patients were excluded from this analysis if they did not have data related to LOS.

IQR, interquartile range.

### Trends in Hospital Procedures for Simple Extractions and Oral Abscesses

**Figure 2.**
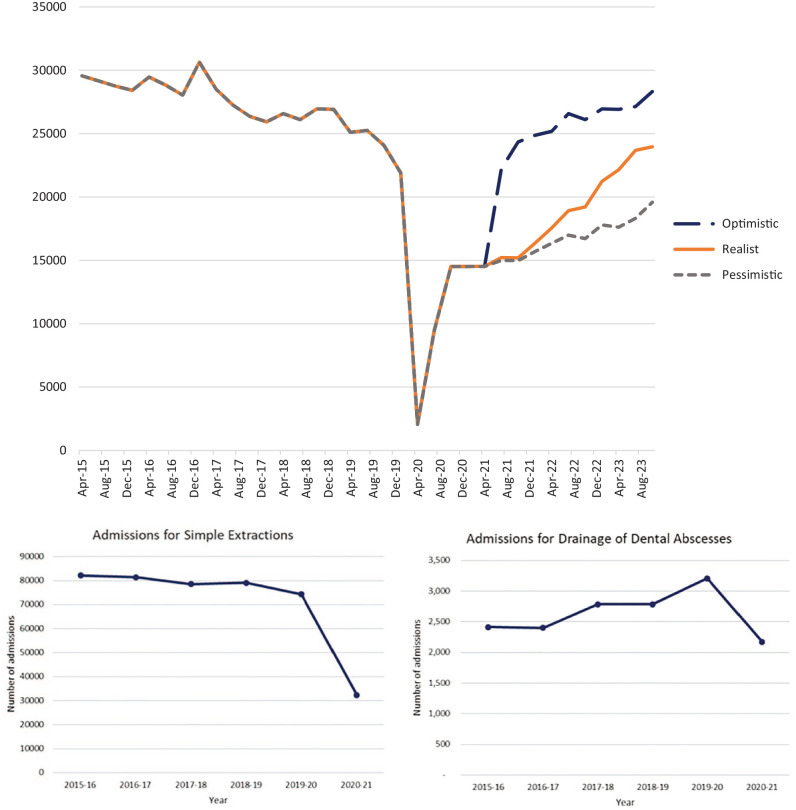
Operative frequency for procedures against time stratified by procedure type. Observed and predicted surgical activity for surgical extractions in hospitals. From October 2020 onward, the frequency of surgical extractions has been modeled. An optimistic, realist, and pessimistic outlook has been presented. For simple extractions and drainage of dental abscesses, only known data are plotted.

Hospital admission data cover 6 financial years, from 2015 to 2021. In total, 439,319 admissions were identified. Of these, 423,541 (96.4%) were for the simple extraction of teeth and 15,778 (3.6%) for drainage of abscess of alveolus of tooth. During the historic period, the median number of simple extractions per year was 80,289 (interquartile range [IQR], 78,839–81,837), and the median number of drainages of dental abscesses was 2,601 (IQR, 2,409–2,788). In comparison, during the years affected by COVID-19, the median number of simple extractions per year was 53,331 (IQR, 32,336–74,326), a 34% reduction (Fig. 2). The median number of drainages of dental abscesses per year during the COVID-19 period was 2,693 (IQR, 2,175–3210), representing a 4% increase. Most of this increase was seen in the year 2019–2020, when only a small proportion of the year was affected by COVID-19; during this year, 3,210 procedures were completed in comparison to 2,175 in 2020–2021. Similar trends were seen when data were stratified by admission category (Appendix 2).

### Trends in Dental Activity in Primary Care

Dental activity within primary care was included from 2014 through 2022. In total, 274,878,400 courses of treatment were identified; 155,281,552 (56%) of these were band 1, 75,169,248 (27%) of these were band 2, 14,042,018 (5%) of these were band 3, 1,058,120 (0.4%) were classified as other, and 29,327,452 (11%) were urgent courses of treatment. A total of 195,053,536 of these courses of treatment were for adults, 49,605,392 (18%) nonpaying and 145,448,144 (53%) paying, and 79,824,840 courses of treatment were for children (29%). All courses of treatment saw a reduction in activity during the COVID-19 pandemic that did not return to prepandemic levels by 2022, as seen in Appendix 4, except for urgent courses of treatment.

### Dental Workforce

The dental workforce, based on dentists working in primary care, ranged from 23,733 to 24,684 during the duration of the study, with a mean (SD) of 24,198 (316.64) dentists working in primary care (CI, 23,933–24,463). A decrease of 4% was seen following COVID-19, with 24,684 dentists working in 2018–2019 and 23,733 in 2020–2021. There was some recovery, with 24,272 dentists working in the year 2021–2022.

### Impact of COVID-19 on Surgical Extractions in Hospitals

During the historic period, 2015–2019, there was a total of 521,972 cases identified, with a median of 111,987 admissions for surgical extractions per year (IQR, 106,569–116,449). During the months affected by COVID-19, 47,966 cases were identified. The median number of surgical extractions per year decreased to 61,204 (IQR, 26,059–96,349) during 2020–2021. The demographics saw little variation when comparing pre–COVID-19 frequencies to those seen during the pandemic (Table 2, Appendix 3). There was a median of 27,256 admissions per quarter (IQR, 26,108–28,825) in the historic period and a median of 12,003 admissions per quarter in the COVID-19 period, starting in January 2020 (IQR, 5,769–18,215) (Table 3). This indicates a 56% reduction in surgical activity. The total cumulative deficit of hospital admissions for surgical extractions procedures up to December 31, 2021, was approximately 61,058 procedures. Similar trends were seen when the data were stratified by age and admission category (Appendix 5). The modeled volume of surgical activity in 2021–2023 suggests that surgical extraction activity has not returned to prepandemic levels (Fig. 2).

**Table 3.** Frequency of Surgical Extractions, as Defined by Code F09.

Year	Quarter	Admissions for Surgical Extractions, *n* (CI)	Mean Admission per Quarter
2015–2016	1	29,563 (11,297–47,830)	—
	2	29,162 (11,536–46,788)	—
	3	28,757 (11,186–46,328)	—
	4	28,428 (11,127–45,730)	28,977.5
2016–2017	1	29,470 (11,409–47,531)	—
	2	28,825 (11,555–46,095)	—
	3	28,045 (10,966–45,124)	—
	4	30,648 (11,860–49,437)	29,247.0
2017–2018	1	28,501 (11,036–45,966)	—
	2	27,256 (11,133–43,379)	—
	3	26,366 (10,472–42,260)	—
	4	25,940 (10,489–41,391)	27,015.75
2018–2019	1	26,583 (10,670–42,496)	—
	2	26,108 (10,752–41,464)	—
	3	26,960 (10,653–43,267)	—
	4	26,918 (10,835–43,001)	26,642.25
2019–2020	1	25,110 (10,181–40,039)	—
	2	25,256 (10,368–40,144)	—
	3	24,076 (9,867–38,285)	—
	4	21,907 (8,289–35,525)	24,087.25
2020–2021	1	2,053 (860–3,246)	—
	2	9,484 (3,876–15,092)	—
	3	14,522 (6,154–22,890)	8,686.33
Modeled number of surgical extraction procedures			
	4	14,522	10,145.25
2021–2022	1	14,522	—
	2	15,211	—
	3	15,189	—
	4	16,344	15316.5
2022–2023	1	17,553	—
	2	18,521	—
	3	19,215	—
	4	21,234	19,297.75
2023–2024	1	22,151	—
	2	23,684	—
	3	23,962	23,265.67

Results are stratified by quarters and a mean admission per quarter presented for each year. Modeled numbers of procedures are displayed for 2020 to 2021 (quarter 1) until 2023 to 2024 (quarter 3).

CI, confidence interval.

## Discussion

The principal finding of this observational study is that there were 8,686 surgical extractions taking place per quarter in England during the COVID-19 pandemic, representing a 56% reduction in comparison to pre–COVID-19 levels. This corresponds to the deferral of 61,058 surgical extractions in hospitals during the study period. These findings support those of a previous study reporting a 33.6% reduction in surgical activity during the pandemic but indicates that surgical extractions in hospitals were more severely affected than other surgical procedures (Dobbs et al. 2021). The reduction in activity in hospitals was matched by a reduction in general practice activity. These data are temporally associated with the postponement of routine dental activity and reports of patients challenging to access dental care (Witton et al. 2021).

The reduction in nonelective cases may be due to a decrease in the number of patients presenting to the emergency department with dental pain or abscesses and uncertainty as to the safety of delivering surgical care during the pandemic (Fowler, Abbott, et al. 2021). It was reported that during COVID-19, there was a reduction in patient attendance to emergency departments for hypothesized reasons, such as hospital avoidance behaviors due to the public fear of contracting COVID-19 (Reschen et al. 2021; Vollmer et al. 2021) and successful provision of emergency dental treatment in UDCs (Politi et al. 2020). Anecdotally, while academic papers and media outlets have mentioned a reduction in dental activity during the COVID-19 pandemic, this study provides a nationwide complete data set that reports the true impact of the pandemic on the volume of surgical extractions being conducted in hospitals. Our data reflect the true reduction in surgical extractions in hospital settings. This reduction occurred for several reasons, such as infection risks associated with aerosol-generated procedures, including the use of surgical handpieces for extractions (Watt 2020), redeployment of oral surgery teams, and the utilization of surgical space for intensive care units (Winterburn 2021). The findings of this study reflect the national guidance given that recommended the suspension of nonurgent procedures such as asymptomatic teeth. This was to ensure PPE, staff, and hospital capacity were available to treat patients who were seriously ill because of COVID-19 (British Association of Oral and Maxillofacial Surgeons 2020).

It was anticipated that number of patients attending the hospital with dental abscesses would increase because of postponed dental care. This effect was not seen in this cohort. This study is unable to capture the true impact of delayed care as data only cover up to December 2020 and do not reflect the totality of the recovery period. There is some limited evidence that postponed dental care has resulted in delays in oral cancer diagnosis (Sandhu et al. 2021), patients self-treating dental conditions (Westgarth 2021b), and increases in dental infections (Dave et al. 2020; Yakubov et al. 2020). As dental services recover, uptake has been slowest for older adults, children, and those in higher deprivation profiles, who are the most affected by access challenges (Stennett and Tsakos 2022). While this study is situated in England, it is likely that other countries will have seen similar reductions in dental activity during the COVID-19 pandemic. This substantial reduction in dental activity will have undoubtedly had significant impacts globally.

This analysis has several strengths. First, this observational cohort study included data from all patients who were admitted for a surgical extraction of a tooth in England in the months leading up to and during the COVID-19 pandemic. The results represent an accurate picture of surgical extractions and associated decrease in activity during the pandemic. Second, over half a million surgical extractions were identified and included, making this the largest observational study in England relating to surgical extractions that the authors are aware of. Surgical extractions taking place in the private sector on the NHS were also included in the analysis to capture activity that was redirected to these facilities in a bid to reduce the impact of COVID-19 (NHS England 2020). Last, the context of this study was nested in publicly available data to provide context to the trends seen for surgical extraction procedures in hospitals.

This study has certain limitations. The impact of COVID-19 on dental activity in hospitals was determined predominantly by the number of surgical extraction procedures taking place. Neglecting other dental treatments delivered in hospitals makes it challenging to draw conclusions as to the complete impact of COVID-19 on service provision. Surgical extractions are classified as AGPs due to the use of a surgical handpiece, whereas simple extractions are non-AGPs. It is likely a greater reduction was seen in surgical extractions in comparison to simple extractions for this reason and concerns over the higher risk of COVID-19 transmission (Virdi et al. 2021). To overcome this, we intentionally included data from NHS Digital for simple extractions, which showed a similar reduction in activity, suggesting service reduction was universal. This quantitative assessment of the impact of COVID-19 does not assess the change in severity of dental abscesses presenting during the pandemic. One study found that while referrals to the emergency department for dental abscesses decreased, the proportion of these that required admission increased (Long and Corsar 2020). The duration of study is limited to December 2020; therefore, the true impact of the COVID-19 pandemic is not shown. Instead, a model has been presented based on estimated activity. There is the need for further analysis of updated HES data to determine whether this corresponds to the modeled scenario. The limitation in study duration is particularly relevant for nonelective admissions due to dental abscesses possibly seen due to delayed care. Furthermore, the impact on NHS waiting lists is not shown, and further studies are needed to indicate the longer-term impacts of the pandemic. Another consideration is whether the number of admissions is a comparative measure when looking at admissions prior to and during the pandemic. It is possible this study underestimates the number of surgical procedures during the pandemic as activity was purposely shifted to community settings and some patients may have opted to have treatment privately as access to NHS treatment was limited. Both settings would not be captured with these data. These data presented do not provide information as to how many teeth were symptomatic, the reason for referral to a hospital setting, or the clinical reason an extraction was required, only the admission category (Appendix 2). Further explorative research is needed to better understand the processes occurring within this data set to inform future service planning.

Moreover, extraction of decayed teeth is the most common reason for children aged 5 to 9 y to require a general anesthetic in the United Kingdom. With this consideration, research is needed with a focus on pediatric cases to determine the exact number of children undergoing dental extractions (Royal College of Surgeons 2015).

## Conclusion

In conclusion, this study quantifies the impact of COVID-19 on the provision of surgical extractions in hospital settings. Further research is needed to determine the impact of COVID-19 on other dental procedures and the long-term implications of this delayed care.

## Author Contributions

J. Booth, T.E.F. Abbott, contributed to conception, design, data analysis and interpretation, drafted and critically revised the manuscript; A.J. Fowler, contributed to conception, data acquisition and analysis, critically revised the manuscript; R. Pearse, contributed to conception, design, data acquisition, critically revised the manuscript; P. Dias, contributed to data acquisition, critically revised the manuscript; Y.I. Wan, contributed to design, data interpretation, critically revised the manuscript; R. Witton, contributed to design, data interpretation, drafted and critically revised the manuscript. All authors have their final approval and agree to be accountable for all aspects of work.

## Supplemental Material

sj-docx-1-jct-10.1177_23800844231216356 – Supplemental material for Dental Surgical Activity in Hospitals during COVID-19: A Nationwide Observational Cohort Study Supplemental material, sj-docx-1-jct-10.1177_23800844231216356 for Dental Surgical Activity in Hospitals during COVID-19: A Nationwide Observational Cohort Study by J. Booth, A.J. Fowler, R. Pearse, P. Dias, Y.I. Wan, R. Witton and T.E.F. Abbott in JDR Clinical & Translational Research
