# Supplementary material for: Dental Surgical Activity in Hospitals during COVID-19: A Nationwide Observational Cohort Study
Source: JDR Clin Trans Res. 2024 Jan 3;9(4):387–97. doi: 10.1177/23800844231216356 (PMC11409558; doi:10.1177/23800844231216356)
Supplement: sj-docx-1-jct-10.1177_23800844231216356 – Supplemental material for Dental Surgical Activity in Hospitals during COVID-19: A Nationwide Observational Cohort Study [file sj-docx-1-jct-10.1177_23800844231216356.docx]

**Dental surgical activity in hospitals: a nationwide observational cohort study in England**

**Supplement**

Joelle Booth^1,2^, Alexander J Fowler^3^, Rupert Pearse^3^, Priya Dias^3^, Yize Wan^3^, Robert Witton^2^, Tom Abbott^3^

1. Dental Public Health and Primary Care, Barts & The London School of Medicine and Dentistry, Queen Mary University of London, London, UK
2. Peninsula Dental School, University of Plymouth, Drake Circus, Plymouth, UK
3. Critical Care and Perioperative Medicine Research Group, William Harvey Research Institute, Queen Mary University of London, London, UK

**S1 – Urgent Dental Care Patient Pathway. Diagram taken from NHS England**


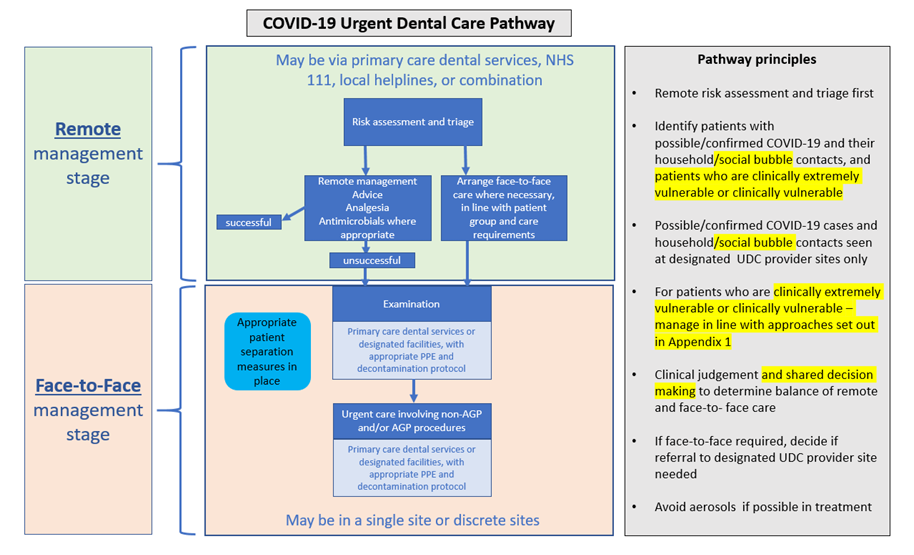

NHS England. COVID-19 guidance and standard operating procedure: For the provision of urgent dental care in primary care dental settings and designated urgent dental care provider sites. 2020. Available at https://www.england.nhs.uk/coronavirus/wp-content/uploads/sites/52/2020/06/C0581-covid-19-urgent-dental-care-sop-update-16-june-20-.pdf (accessed October 2023)

**S2 – Hospital Episode Statistics Admitted Patient Care Data Dictionary Elective and Non-elective Admissions**

| **Elective Admission** | **Non-elective Admissions** |
| --- | --- |
| Waiting list – a patient admitted electively from a waiting list | Emergency admissions - when admission is unpredictable and at short notice due to clinical need such as admission from the emergency department |
| Booked – a patient admitted having been given a date at the time of the decision to admit was made | Other - Transfer of any admitted patient from other Hospital Provider among other admission routes |
| Planned **–** a patient admitted having been given a date or approximate date at the time that the decision to admit was made |  |

NHS Digital. Hospital Episode Statistics Data Dictionary. 2023. Available at https://digital.nhs.uk/data-and-information/data-tools-and-services/data-services/hospital-episode-statistics/hospital-episode-statistics-data-dictionary (accessed October 2023)

**S3 – Surgical extractions stratified by Index of Multiple Deprivation during the pre-COVID and COVID period.**

**
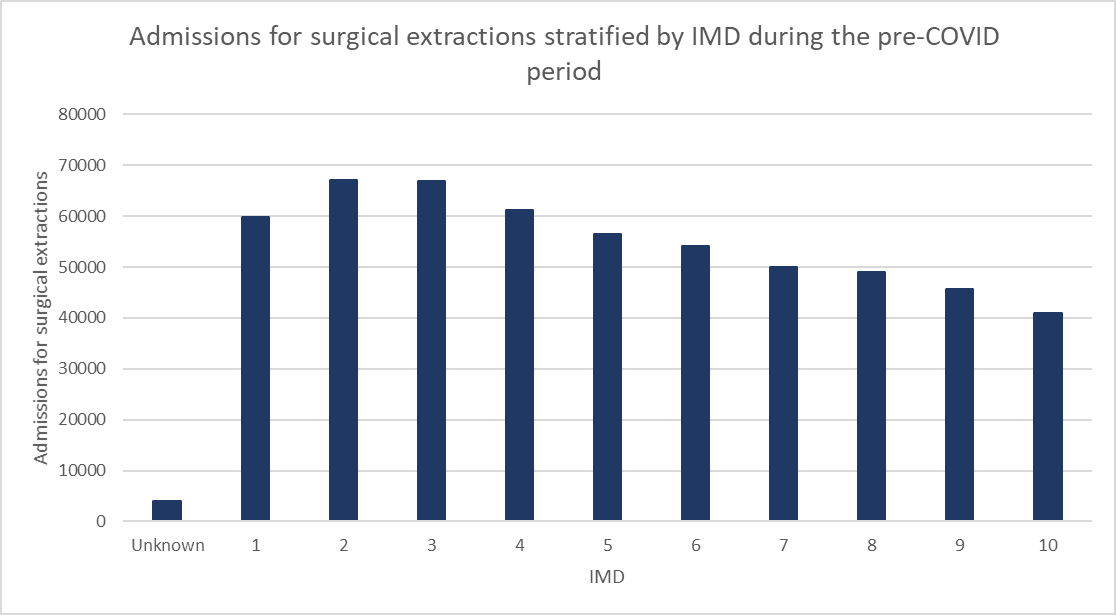


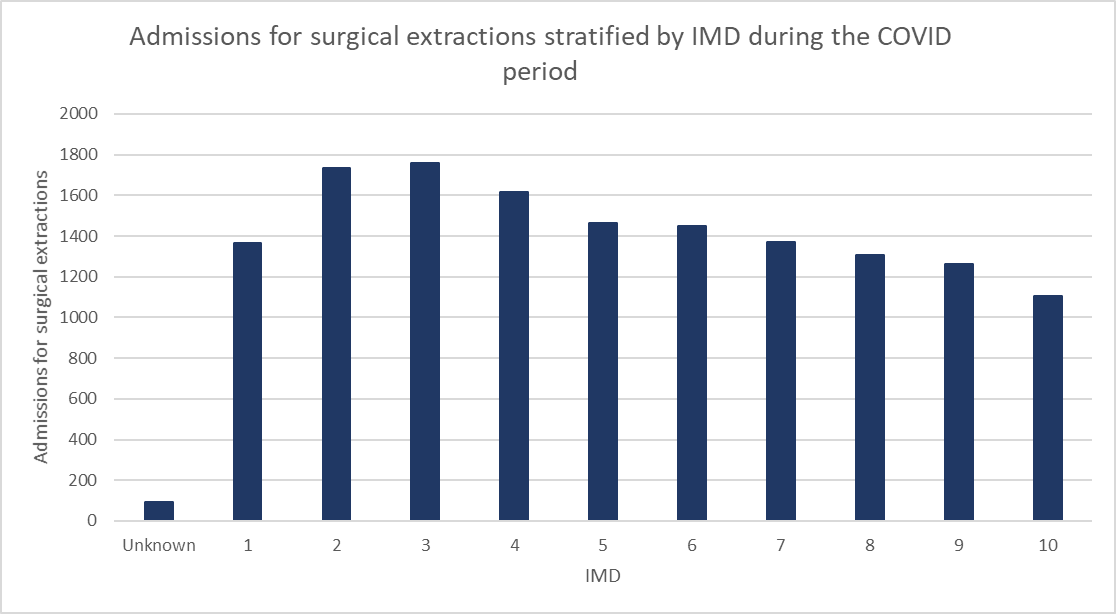
**

**S4 - Primary dental care activity stratified by course of treatment (COT) type, band 1, band 2, band 3 and urgent.**

**
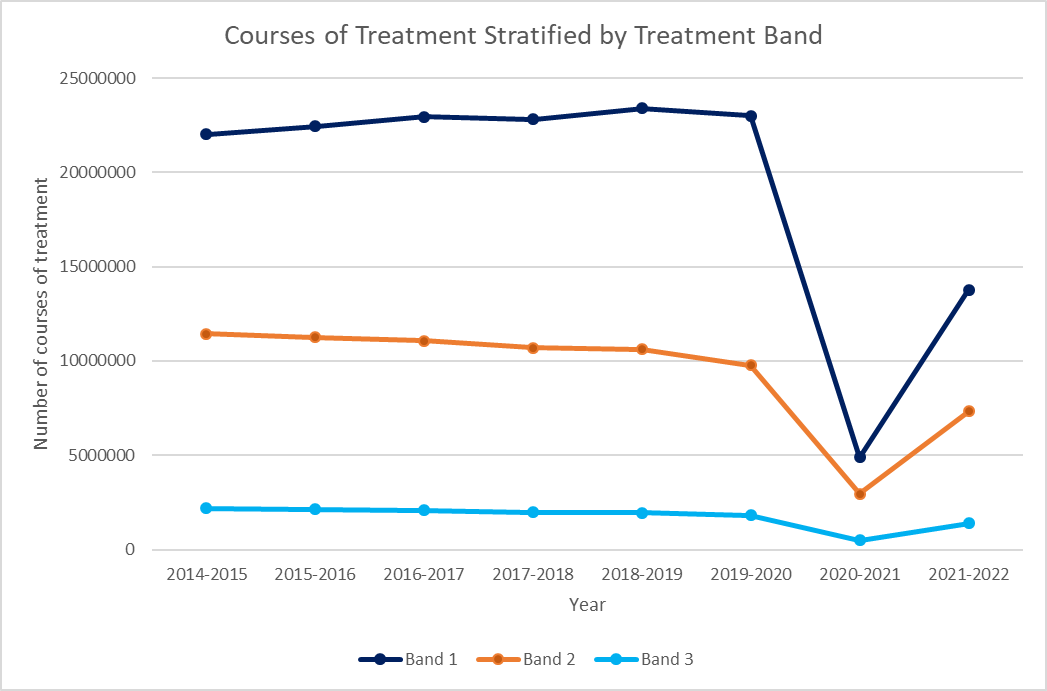

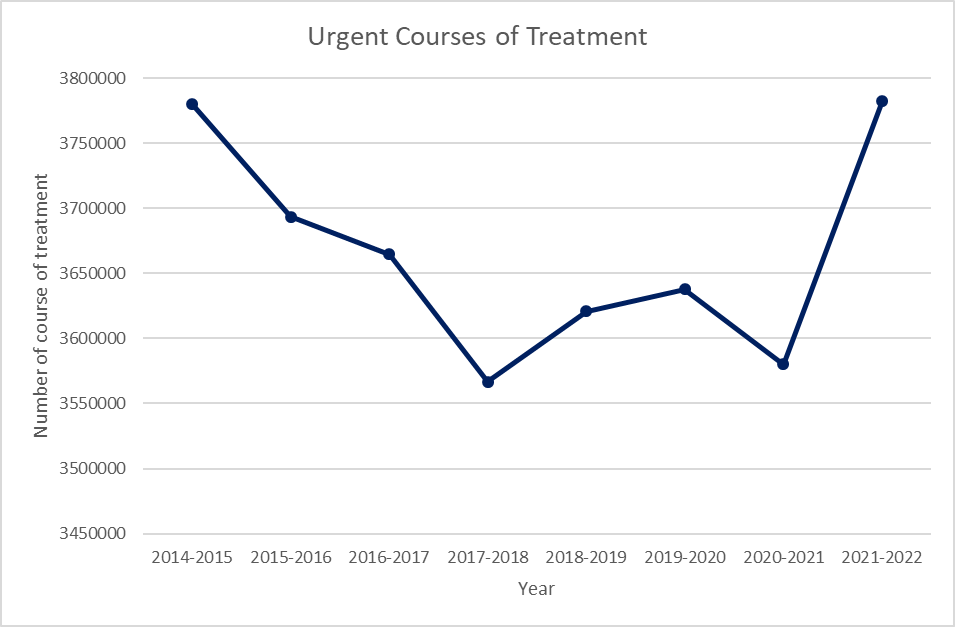
**

**S5 – Surgical extraction admissions stratified by sex, admission category and age.**

**
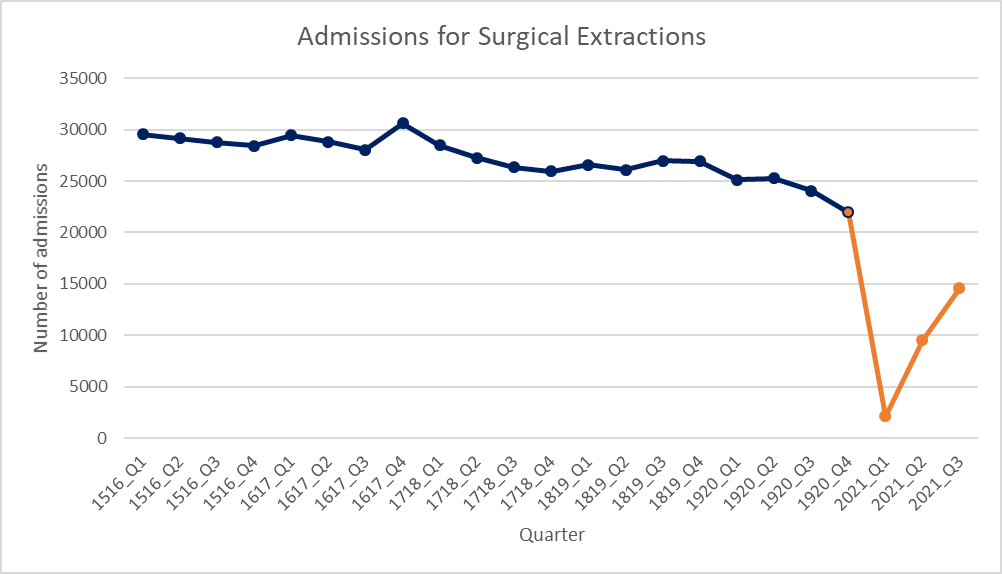
**

**
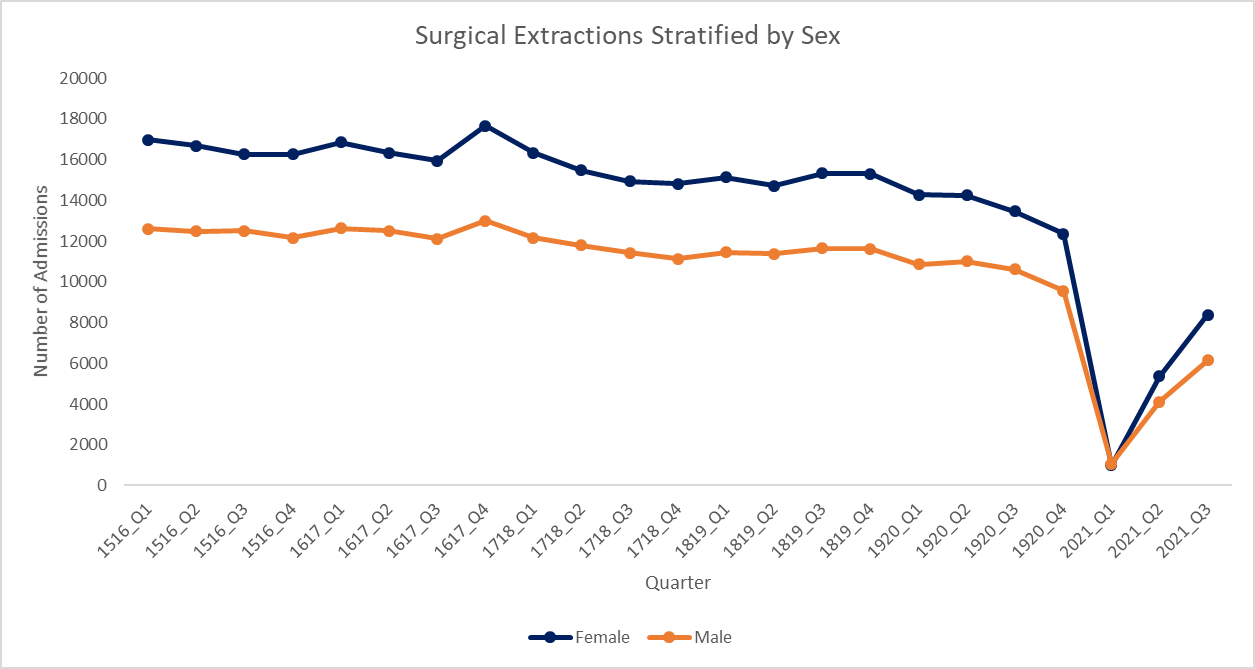
**

**
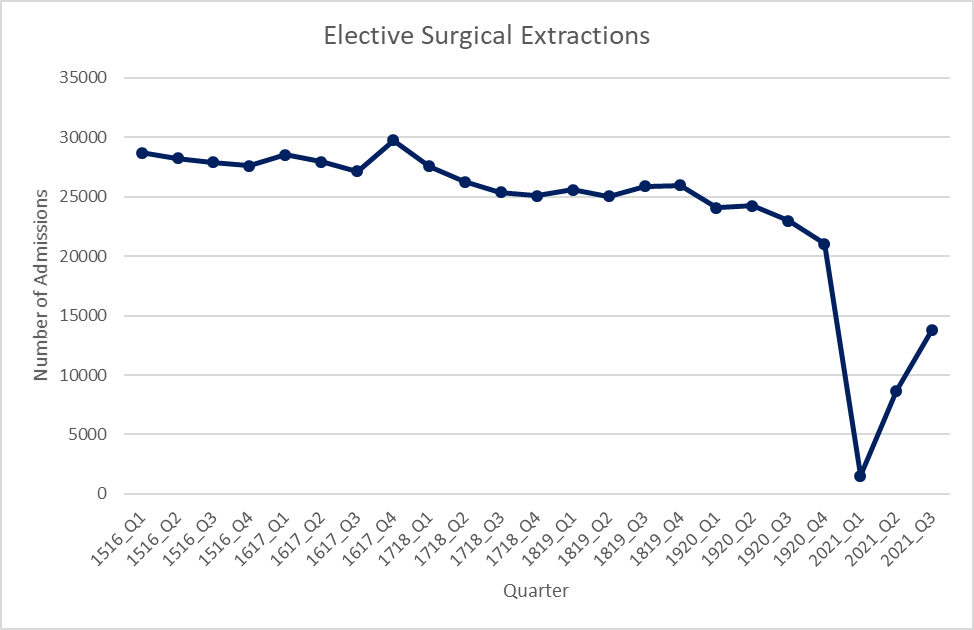
**

**
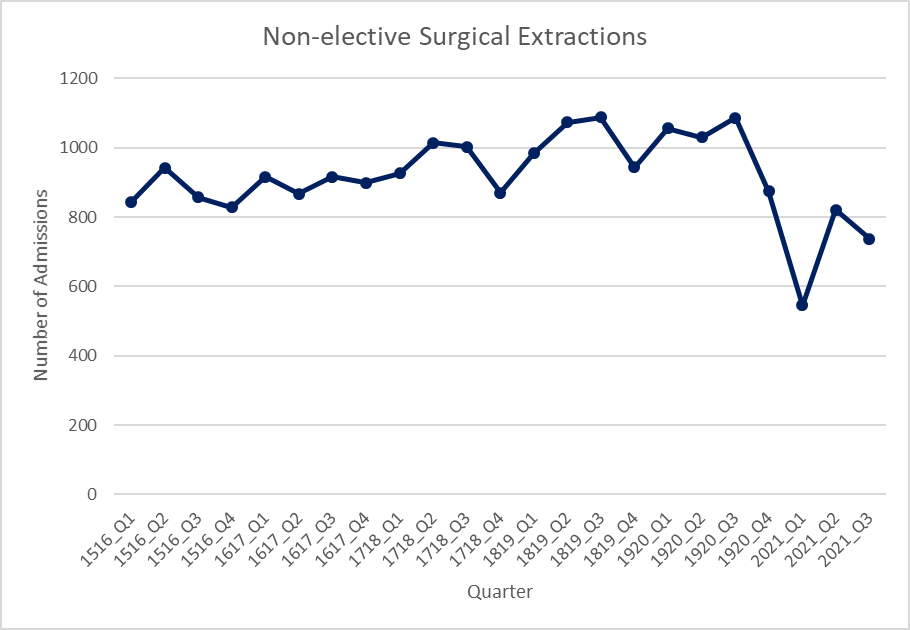
**

**
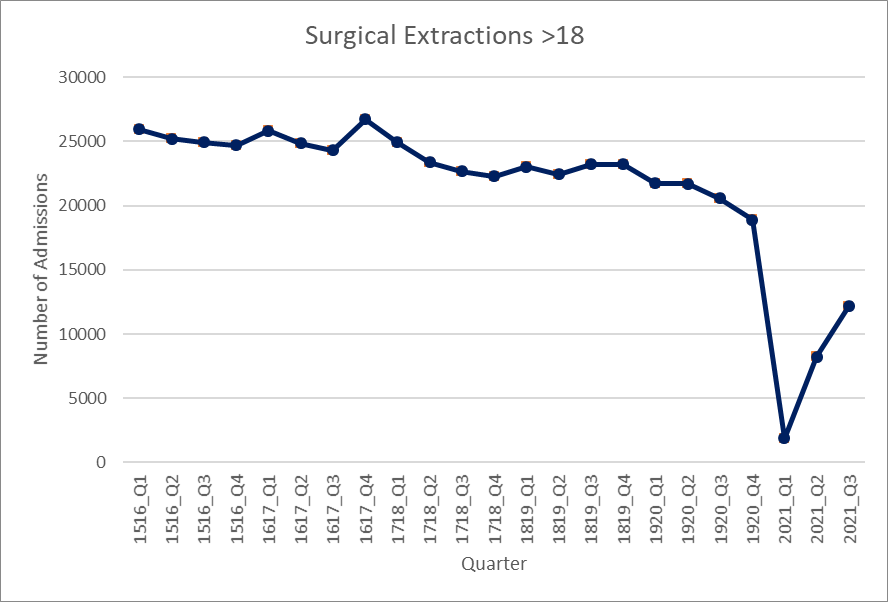
**

**
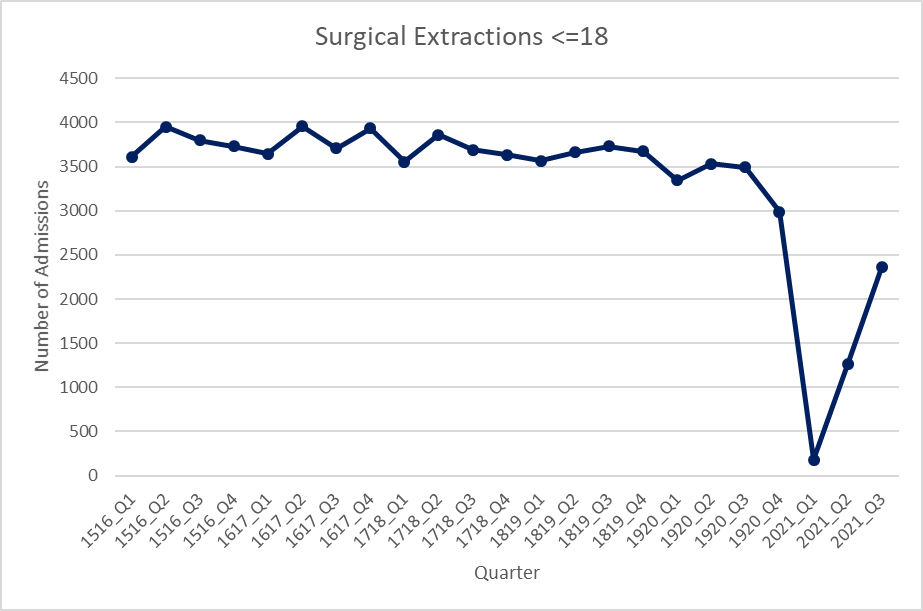
**
